# {2-[(3-Bromo­benzyl­idene)amino]-5-chloro­phen­yl}(phen­yl)methanone

**DOI:** 10.1107/S1600536812004667

**Published:** 2012-02-10

**Authors:** M. Aslam, I. Anis, N. Afza, M. Safder, S. Yousuf

**Affiliations:** a Pharmaceutical Research Centre, PCSIR Laboratories Complex, Karachi, Pakistan; b Department of Chemistry, University of Karachi, Karachi, Pakistan; c HEJ Research Institute of Chemistry, International Center for Chemical and Biological Sciences, University of Karachi, Karachi 75270, Pakistan

## Abstract

In the title compound, C_20_H_13_BrClNO, the azomethine double bond [C=N = 1.246 (4) Å] adopts an *E* conformation. The bromo- and chlorophenyl rings are inclined to one another by 13.70 (11)°, and form dihedral angles of 76.68 (10) and 74.24 (7)°, respectively, with the phenyl ring. In the crystal, mol­ecules are linked by C—H⋯O hydrogen bonds to form double stranded chains propagating along the *b*-axis direction.

## Related literature

For background information and preparation of Schiff bases, see: Khan *et al.* (2009 ▶); Aslam *et al.* (2011*a* ▶,*b* ▶); Zeb & Yousuf (2011 ▶). For the crystal structures of related Schiff bases, see: Aslam *et al.* (2011*a* ▶,*b* ▶); Cox *et al.* (2008 ▶).
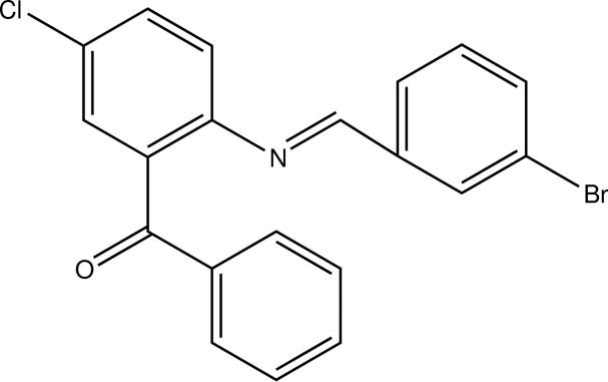


## Experimental

### 

#### Crystal data

C_20_H_13_BrClNO
*M* *_r_* = 398.67
Orthorhombic, 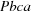

*a* = 16.2068 (12) Å
*b* = 7.8839 (6) Å
*c* = 27.262 (2) Å
*V* = 3483.4 (5) Å^3^
*Z* = 8
Mo *K*α radiation
μ = 2.52 mm^−1^
*T* = 273 K
0.52 × 0.21 × 0.15 mm

#### Data collection

Bruker SMART APEX CCD area-detector diffractometer
Absorption correction: multi-scan (*SADABS*; Bruker, 2000 ▶) *T*_min_ = 0.354, *T*_max_ = 0.704
19261 measured reflections
3243 independent reflections
1931 reflections with *I* > 2σ(*I*)
*R*_int_ = 0.056

#### Refinement

*R*[*F* ^2^ > 2σ(*F* ^2^)] = 0.045
*wR*(*F* ^2^) = 0.115
*S* = 1.01
3243 reflections
217 parameters
H-atom parameters constrained
Δρ_max_ = 0.40 e Å^−3^
Δρ_min_ = −0.64 e Å^−3^

### 

Data collection: *SMART* (Bruker, 2000 ▶); cell refinement: *SAINT* (Bruker, 2000 ▶); data reduction: *SAINT*; program(s) used to solve structure: *SHELXS97* (Sheldrick, 2008 ▶); program(s) used to refine structure: *SHELXL97* (Sheldrick, 2008 ▶); molecular graphics: *SHELXTL* (Sheldrick, 2008 ▶); software used to prepare material for publication: *SHELXTL*, *PARST* (Nardelli, 1995 ▶) and *PLATON* (Spek, 2009 ▶).

## Supplementary Material

Crystal structure: contains datablock(s) global, I. DOI: 10.1107/S1600536812004667/pv2506sup1.cif

Structure factors: contains datablock(s) I. DOI: 10.1107/S1600536812004667/pv2506Isup2.hkl

Supplementary material file. DOI: 10.1107/S1600536812004667/pv2506Isup3.cml

Additional supplementary materials:  crystallographic information; 3D view; checkCIF report

## Figures and Tables

**Table 1:** Hydrogen-bond geometry (Å, °)

*D*—H⋯*A*	*D*—H	H⋯*A*	*D*⋯*A*	*D*—H⋯*A*
C12—H12*A*⋯O1^i^	0.93	2.44	3.346 (4)	166
C17—H17*A*⋯O1^ii^	0.93	2.51	3.428 (5)	168

Symmetry codes: (i) 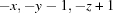; (ii) 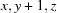.

**Fig. 1.**
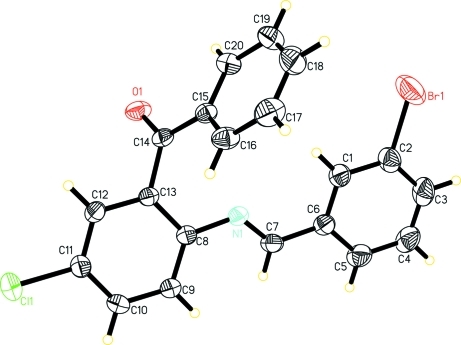
The molecular structure of the title compound with the atom numbering scheme. Displacement ellipsoids are drawn at the 30% probability level. H atoms are presented as small spheres of arbitrary radius.

**Fig. 2.**
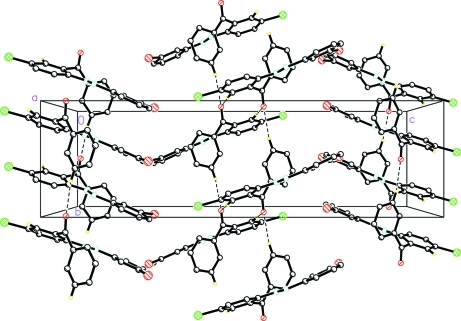
A view of the C—-H···O hydrogen bonds (dotted lines) in the crystal structure of the title compound. H atoms non-participating in hydrogen- bonding were omitted for clarity.

## supplementary crystallographic information

### Comment

The title compound was prepared as a part of our ongoing reasearch on schiff bases (Khan *et al.*, 2009; Aslam *et al.*, 2011*a*,*b*; Zeb & Yousuf, 2011).

In the title compound (Fig. 1), the azomethine double bond (C═N, 1.246 (4) Å) adopts an E configuration with torsion angle C6—C7—N1—C8 174.9 (3)°. The bond lengths and angle are similar as in other structurally realted compounds (Aslam *et al.*, 2011*a*,*b*; Cox *et al.*, 2008). In the crystal structure the molecules are arranged in parallel sheets along the *b*-axis *via* C—H···O type intermolecular hydrogen bonds (Fig. 2).

### Experimental

A mixture of 3-bromobenzaldehyde (1 mol) and 2-amino-5-chlorobenzophenone (1 mol) in ethanol (50 ml) along with 3 drops of conc. H_2_SO_4_ was refluxed for 5 h at 343 K. After cooling, the mixture was concentrated to one third under reduced pressure. The concentrated reaction mixture was kept at room temperature and orange red crystals were obtained after five days. The crystalline product was collected, washed with methanol and dried to afford the title compound in 87% yield. Slow evaporation of a methanol solution afforded yellow crystals suitable for single-crystal X-ray diffraction studies. All chemicals were purchased from Sigma-Aldrich.

### Refinement

H atoms were positioned geometrically with C—H = 0.93 Å, and constrained to ride on their parent atoms with *U*_iso_(H) = 1.2*U*_eq_(C).

### Crystal data

**Table:**

C_20_H_13_BrClNO	*F*(000) = 1600
*M**_r_* = 398.67	*D*_x_ = 1.520 Mg m^−^^3^
Orthorhombic, *P**b**c**a*	Mo *K*α radiation, λ = 0.71073 Å
Hall symbol: -P 2ac 2ab	Cell parameters from 2145 reflections
*a* = 16.2068 (12) Å	θ = 2.5–20.5°
*b* = 7.8839 (6) Å	µ = 2.52 mm^−^^1^
*c* = 27.262 (2) Å	*T* = 273 K
*V* = 3483.4 (5) Å^3^	Block, yellow
*Z* = 8	0.52 × 0.21 × 0.15 mm

### Data collection

**Table:**

Bruker SMART APEX CCD area-detector diffractometer	1931 reflections with *I* > 2σ(*I*)
Radiation source: fine-focus sealed tube	*R*_int_ = 0.056
ω scan	θ_max_ = 25.5°, θ_min_ = 1.5°
Absorption correction: multi-scan (*SADABS*; Bruker, 2000)	*h* = −19→19
*T*_min_ = 0.354, *T*_max_ = 0.704	*k* = −9→9
19261 measured reflections	*l* = −33→33
3243 independent reflections	

### Refinement

**Table:**

Refinement on *F*^2^	Primary atom site location: structure-invariant direct methods
Least-squares matrix: full	Secondary atom site location: difference Fourier map
*R*[*F*^2^ > 2σ(*F*^2^)] = 0.045	Hydrogen site location: inferred from neighbouring sites
*wR*(*F*^2^) = 0.115	H-atom parameters constrained
*S* = 1.01	*w* = 1/[σ^2^(*F*_o_^2^) + (0.0377*P*)^2^ + 3.0254*P*] where *P* = (*F*_o_^2^ + 2*F*_c_^2^)/3
3243 reflections	(Δ/σ)_max_ < 0.001
217 parameters	Δρ_max_ = 0.40 e Å^−^^3^
0 restraints	Δρ_min_ = −0.64 e Å^−^^3^

### Special details

**Table:**

Geometry. All e.s.d.'s (except the e.s.d. in the dihedral angle between two l.s. planes) are estimated using the full covariance matrix. The cell e.s.d.'s are taken into account individually in the estimation of e.s.d.'s in distances, angles and torsion angles; correlations between e.s.d.'s in cell parameters are only used when they are defined by crystal symmetry. An approximate (isotropic) treatment of cell e.s.d.'s is used for estimating e.s.d.'s involving l.s. planes.
Refinement. Refinement of *F*^2^ against ALL reflections. The weighted *R*-factor *wR* and goodness of fit *S* are based on *F*^2^, conventional *R*-factors *R* are based on *F*, with *F* set to zero for negative *F*^2^. The threshold expression of *F*^2^ > σ(*F*^2^) is used only for calculating *R*-factors(gt) *etc*. and is not relevant to the choice of reflections for refinement. *R*-factors based on *F*^2^ are statistically about twice as large as those based on *F*, and *R*- factors based on ALL data will be even larger.

### Fractional atomic coordinates and isotropic or equivalent isotropic displacement parameters (Å^2^)

**Table:**

	*x*	*y*	*z*	*U*_iso_*/*U*_eq_	
Br1	0.08907 (4)	0.01989 (9)	0.238025 (19)	0.1197 (3)	
Cl1	0.20842 (7)	−0.43108 (16)	0.61666 (4)	0.0784 (4)	
O1	0.04701 (14)	−0.5034 (3)	0.44146 (10)	0.0608 (7)	
N1	0.21723 (16)	−0.2133 (4)	0.40923 (10)	0.0480 (7)	
C1	0.1977 (2)	−0.0784 (5)	0.31387 (12)	0.0564 (10)	
H1B	0.1496	−0.1092	0.3302	0.068*	
C2	0.1939 (3)	−0.0077 (5)	0.26789 (13)	0.0657 (11)	
C3	0.2627 (3)	0.0425 (5)	0.24302 (14)	0.0756 (13)	
H3A	0.2585	0.0912	0.2121	0.091*	
C4	0.3383 (3)	0.0196 (6)	0.26466 (15)	0.0806 (14)	
H4A	0.3859	0.0535	0.2484	0.097*	
C5	0.3439 (2)	−0.0533 (6)	0.31041 (14)	0.0720 (12)	
H5A	0.3955	−0.0691	0.3246	0.086*	
C6	0.2739 (2)	−0.1034 (5)	0.33568 (12)	0.0508 (9)	
C7	0.2795 (2)	−0.1732 (5)	0.38504 (12)	0.0517 (9)	
H7A	0.3314	−0.1883	0.3990	0.062*	
C8	0.22119 (19)	−0.2687 (4)	0.45845 (11)	0.0421 (8)	
C9	0.2910 (2)	−0.2620 (5)	0.48817 (12)	0.0501 (9)	
H9A	0.3406	−0.2225	0.4753	0.060*	
C10	0.2873 (2)	−0.3135 (5)	0.53632 (12)	0.0534 (9)	
H10A	0.3342	−0.3089	0.5559	0.064*	
C11	0.2143 (2)	−0.3715 (5)	0.55527 (12)	0.0508 (9)	
C12	0.1447 (2)	−0.3843 (4)	0.52657 (12)	0.0485 (9)	
H12A	0.0960	−0.4277	0.5396	0.058*	
C13	0.14765 (18)	−0.3321 (4)	0.47808 (11)	0.0400 (8)	
C14	0.07325 (18)	−0.3588 (5)	0.44606 (11)	0.0431 (8)	
C15	0.03203 (19)	−0.2139 (4)	0.42183 (11)	0.0427 (8)	
C16	0.0429 (2)	−0.0504 (5)	0.43778 (14)	0.0581 (10)	
H16A	0.0776	−0.0286	0.4642	0.070*	
C18	−0.0479 (3)	0.0481 (6)	0.37522 (18)	0.0799 (13)	
H18A	−0.0746	0.1369	0.3593	0.096*	
C19	−0.0588 (2)	−0.1138 (6)	0.35928 (15)	0.0706 (12)	
H19A	−0.0930	−0.1352	0.3326	0.085*	
C17	0.0027 (2)	0.0814 (6)	0.41493 (17)	0.0750 (12)	
H17A	0.0096	0.1920	0.4261	0.090*	
C20	−0.0199 (2)	−0.2447 (5)	0.38228 (13)	0.0553 (9)	
H20A	−0.0281	−0.3553	0.3714	0.066*	

### Atomic displacement parameters (Å^2^)

**Table:**

	*U*^11^	*U*^22^	*U*^33^	*U*^12^	*U*^13^	*U*^23^
Br1	0.1021 (4)	0.1846 (7)	0.0725 (4)	0.0117 (4)	−0.0213 (3)	0.0344 (4)
Cl1	0.0843 (7)	0.0996 (9)	0.0513 (5)	−0.0078 (6)	−0.0055 (5)	0.0208 (6)
O1	0.0485 (14)	0.0481 (16)	0.0857 (19)	−0.0091 (13)	−0.0129 (13)	−0.0014 (13)
N1	0.0384 (15)	0.059 (2)	0.0470 (16)	−0.0057 (14)	0.0019 (13)	0.0018 (14)
C1	0.060 (2)	0.066 (3)	0.043 (2)	−0.005 (2)	0.0067 (17)	−0.0031 (18)
C2	0.081 (3)	0.072 (3)	0.044 (2)	−0.002 (2)	−0.0013 (19)	−0.003 (2)
C3	0.109 (4)	0.077 (3)	0.041 (2)	−0.012 (3)	0.012 (2)	−0.001 (2)
C4	0.087 (4)	0.100 (4)	0.055 (2)	−0.028 (3)	0.024 (2)	−0.005 (2)
C5	0.060 (2)	0.099 (3)	0.056 (2)	−0.016 (2)	0.0112 (19)	−0.006 (2)
C6	0.054 (2)	0.056 (2)	0.0417 (18)	−0.0070 (18)	0.0043 (16)	−0.0089 (17)
C7	0.042 (2)	0.065 (3)	0.048 (2)	−0.0048 (18)	−0.0032 (17)	−0.0059 (18)
C8	0.0406 (18)	0.041 (2)	0.0442 (18)	−0.0010 (16)	−0.0002 (15)	−0.0022 (15)
C9	0.0437 (19)	0.056 (2)	0.051 (2)	−0.0079 (18)	−0.0024 (17)	0.0021 (17)
C10	0.046 (2)	0.063 (3)	0.051 (2)	−0.0029 (18)	−0.0121 (17)	0.0017 (18)
C11	0.055 (2)	0.051 (2)	0.0462 (19)	−0.0017 (19)	−0.0016 (17)	0.0047 (17)
C12	0.0436 (19)	0.049 (2)	0.053 (2)	−0.0058 (17)	0.0027 (16)	0.0057 (17)
C13	0.0356 (18)	0.0359 (19)	0.0483 (19)	−0.0002 (15)	−0.0017 (14)	0.0002 (15)
C14	0.0329 (17)	0.048 (2)	0.0480 (19)	−0.0035 (17)	0.0048 (14)	−0.0056 (17)
C15	0.0356 (17)	0.042 (2)	0.0503 (19)	0.0025 (16)	0.0022 (15)	−0.0020 (16)
C16	0.046 (2)	0.052 (3)	0.077 (3)	0.0043 (19)	−0.0056 (19)	−0.007 (2)
C18	0.066 (3)	0.076 (3)	0.098 (3)	0.016 (3)	−0.003 (3)	0.026 (3)
C19	0.059 (3)	0.084 (3)	0.069 (3)	0.007 (2)	−0.015 (2)	0.011 (2)
C17	0.065 (3)	0.052 (3)	0.109 (3)	0.006 (2)	0.002 (3)	−0.001 (2)
C20	0.050 (2)	0.061 (2)	0.055 (2)	0.0024 (19)	−0.0054 (18)	−0.0049 (19)

### Geometric parameters (Å, º)

**Table:**

Br1—C2	1.896 (4)	C9—H9A	0.9300
Cl1—C11	1.741 (3)	C10—C11	1.369 (5)
O1—C14	1.223 (4)	C10—H10A	0.9300
N1—C7	1.246 (4)	C11—C12	1.377 (4)
N1—C8	1.412 (4)	C12—C13	1.385 (4)
C1—C2	1.373 (5)	C12—H12A	0.9300
C1—C6	1.384 (5)	C13—C14	1.503 (4)
C1—H1B	0.9300	C14—C15	1.479 (5)
C2—C3	1.365 (6)	C15—C16	1.372 (5)
C3—C4	1.371 (6)	C15—C20	1.389 (4)
C3—H3A	0.9300	C16—C17	1.376 (5)
C4—C5	1.376 (6)	C16—H16A	0.9300
C4—H4A	0.9300	C18—C19	1.360 (6)
C5—C6	1.385 (5)	C18—C17	1.383 (6)
C5—H5A	0.9300	C18—H18A	0.9300
C6—C7	1.457 (5)	C19—C20	1.363 (5)
C7—H7A	0.9300	C19—H19A	0.9300
C8—C9	1.393 (4)	C17—H17A	0.9300
C8—C13	1.399 (4)	C20—H20A	0.9300
C9—C10	1.375 (4)		
			
C7—N1—C8	123.0 (3)	C10—C11—C12	121.2 (3)
C2—C1—C6	119.4 (4)	C10—C11—Cl1	120.0 (3)
C2—C1—H1B	120.3	C12—C11—Cl1	118.8 (3)
C6—C1—H1B	120.3	C11—C12—C13	119.4 (3)
C3—C2—C1	122.3 (4)	C11—C12—H12A	120.3
C3—C2—Br1	119.1 (3)	C13—C12—H12A	120.3
C1—C2—Br1	118.6 (3)	C12—C13—C8	120.1 (3)
C2—C3—C4	118.6 (4)	C12—C13—C14	119.0 (3)
C2—C3—H3A	120.7	C8—C13—C14	120.7 (3)
C4—C3—H3A	120.7	O1—C14—C15	121.1 (3)
C3—C4—C5	120.2 (4)	O1—C14—C13	118.0 (3)
C3—C4—H4A	119.9	C15—C14—C13	120.9 (3)
C5—C4—H4A	119.9	C16—C15—C20	119.3 (3)
C4—C5—C6	121.1 (4)	C16—C15—C14	121.7 (3)
C4—C5—H5A	119.5	C20—C15—C14	119.0 (3)
C6—C5—H5A	119.5	C15—C16—C17	120.4 (4)
C1—C6—C5	118.4 (3)	C15—C16—H16A	119.8
C1—C6—C7	120.4 (3)	C17—C16—H16A	119.8
C5—C6—C7	121.1 (3)	C19—C18—C17	120.4 (4)
N1—C7—C6	122.3 (3)	C19—C18—H18A	119.8
N1—C7—H7A	118.9	C17—C18—H18A	119.8
C6—C7—H7A	118.9	C18—C19—C20	120.2 (4)
C9—C8—C13	118.9 (3)	C18—C19—H19A	119.9
C9—C8—N1	125.3 (3)	C20—C19—H19A	119.9
C13—C8—N1	115.8 (3)	C16—C17—C18	119.4 (4)
C10—C9—C8	120.6 (3)	C16—C17—H17A	120.3
C10—C9—H9A	119.7	C18—C17—H17A	120.3
C8—C9—H9A	119.7	C19—C20—C15	120.3 (4)
C11—C10—C9	119.8 (3)	C19—C20—H20A	119.8
C11—C10—H10A	120.1	C15—C20—H20A	119.8
C9—C10—H10A	120.1		
			
C6—C1—C2—C3	−1.4 (6)	C11—C12—C13—C8	0.7 (5)
C6—C1—C2—Br1	178.4 (3)	C11—C12—C13—C14	175.4 (3)
C1—C2—C3—C4	0.7 (6)	C9—C8—C13—C12	1.1 (5)
Br1—C2—C3—C4	−179.0 (3)	N1—C8—C13—C12	−177.9 (3)
C2—C3—C4—C5	0.3 (7)	C9—C8—C13—C14	−173.5 (3)
C3—C4—C5—C6	−0.6 (7)	N1—C8—C13—C14	7.5 (4)
C2—C1—C6—C5	1.0 (6)	C12—C13—C14—O1	−57.2 (4)
C2—C1—C6—C7	178.1 (3)	C8—C13—C14—O1	117.5 (4)
C4—C5—C6—C1	0.0 (6)	C12—C13—C14—C15	121.4 (3)
C4—C5—C6—C7	−177.1 (4)	C8—C13—C14—C15	−63.9 (4)
C8—N1—C7—C6	−174.9 (3)	O1—C14—C15—C16	159.3 (3)
C1—C6—C7—N1	0.3 (6)	C13—C14—C15—C16	−19.3 (5)
C5—C6—C7—N1	177.3 (4)	O1—C14—C15—C20	−19.9 (5)
C7—N1—C8—C9	10.0 (5)	C13—C14—C15—C20	161.6 (3)
C7—N1—C8—C13	−171.1 (3)	C20—C15—C16—C17	0.1 (5)
C13—C8—C9—C10	−1.4 (5)	C14—C15—C16—C17	−179.0 (3)
N1—C8—C9—C10	177.5 (3)	C17—C18—C19—C20	−0.1 (6)
C8—C9—C10—C11	−0.1 (5)	C15—C16—C17—C18	−0.9 (6)
C9—C10—C11—C12	2.0 (6)	C19—C18—C17—C16	0.9 (6)
C9—C10—C11—Cl1	−178.2 (3)	C18—C19—C20—C15	−0.8 (6)
C10—C11—C12—C13	−2.3 (5)	C16—C15—C20—C19	0.7 (5)
Cl1—C11—C12—C13	177.9 (3)	C14—C15—C20—C19	179.9 (3)

### Hydrogen-bond geometry (Å, º)

**Table:**

*D*—H···*A*	*D*—H	H···*A*	*D*···*A*	*D*—H···*A*
C12—H12*A*···O1^i^	0.93	2.44	3.346 (4)	166
C17—H17*A*···O1^ii^	0.93	2.51	3.428 (5)	168

Symmetry codes: (i) −*x*, −*y*−1, −*z*+1; (ii) *x*, *y*+1, *z*.

## References

[bb1] Aslam, M., Anis, I., Afza, N., Nelofar, A. & Yousuf, S. (2011*a*). *Acta Cryst.* E**67**, o3442–o3443. 10.1107/S1600536811048690 PMC3239075 22199923

[bb2] Aslam, M., Anis, I., Afza, N., Nelofar, A. & Yousuf, S. (2011*b*). *Acta Cryst.* E**67**, o3215. 10.1107/S1600536811046162 PMC3238879 22199732

[bb3] Bruker (2000). *SADABS*, *SMART* and *SAINT* Bruker AXS Inc., Madison, Wisconsin, USA.

[bb4] Cox, P. J., Kechagias, D. & Kelly, O. (2008). *Acta Cryst.* B**64**, 206–216. 10.1107/S0108768108000232 18369292

[bb5] Khan, K. M., Khan, M., Ali, M., Taha, M., Rasheed, S., Perveen, S. & Choudhary, M. I. (2009). *Bioorg. Med. Chem.* **17**, 7795–7801. 10.1016/j.bmc.2009.09.028 19837595

[bb6] Nardelli, M. (1995). *J. Appl. Cryst.* **28**, 659.

[bb7] Sheldrick, G. M. (2008). *Acta Cryst.* A**64**, 112–122. 10.1107/S0108767307043930 18156677

[bb8] Spek, A. L. (2009). *Acta Cryst.* D**65**, 148–155. 10.1107/S090744490804362X PMC2631630 19171970

[bb9] Zeb, A. & Yousuf, S. (2011). *Acta Cryst.* E**67**, o2801. 10.1107/S1600536811037846 PMC3201279 22065813

